# Prognostic factors in children and adolescents with differentiated thyroid cancer treated with total thyroidectomy and radioiodine therapy: a retrospective two-center study from China

**DOI:** 10.3389/fendo.2024.1419141

**Published:** 2024-07-22

**Authors:** Congcong Wang, Yutian Li, Guoqiang Wang, Xinfeng Liu, Yingying Zhang, Chenghui Lu, Jiao Li, Na Han, Zenghua Wang, Zengmei Si, Fengqi Li, Gaixia Lu, Renfei Wang, Xufu Wang

**Affiliations:** ^1^ Department of Nuclear Medicine, The Affiliated Hospital of Qingdao University, Qingdao, Shandong, China; ^2^ Department of Radiology, Qingdao Women and Children’s Hospital, Qingdao, Shandong, China; ^3^ Department of Nuclear Medicine, Shanghai Tenth People’s Hospital, Tongji University School of Medicine, Shanghai, China

**Keywords:** children and adolescents, differentiated thyroid cancer, radioiodine therapy, thyroglobulin, therapeutic response, prognosis

## Abstract

**Purpose:**

This two-center study aimed to explore the main prognostic factors affecting the final disease status in children and adolescents with differentiated thyroid cancer (caDTC) following total thyroidectomy and radioiodine therapy (RAIT).

**Materials and methods:**

All caDTC patients from two centers in the period from 2004-2022 were retrospectively included. At the last follow-up, the patients’ disease status was assessed and classified as an incomplete response (IR) or as an excellent or indeterminate response (EIDR). Then, the difference in preablation stimulated thyroglobulin (ps-Tg) levels between the two groups was compared, and the threshold for predicting IR was determined using receiver operating characteristic (ROC) analysis. Moreover, univariate and multivariate analyses were conducted to identify the factors influencing the patients’ ultimate disease outcomes.

**Results:**

A total of 143 patients (98 females, 45 males; median age 16 years) were recruited. After a median follow-up of 42.9 months, 80 patients (55.9%) exhibited an EIDR, whereas 63 patients (44.1%) exhibited an IR. Patients with an IR had significantly greater ps-Tg levels than did those with an EIDR (median ps-Tg 79.2 ng/mL vs. 9.3 ng/mL, p<0.001). The ROC curve showed that ps-Tg ≥20 ng/mL was the most accurate for predicting IR at the last follow-up. According to multivariate analysis, only ps-Tg, T stage and the therapeutic response to initial RAIT were significantly associated with IR.

**Conclusion:**

In caDTC patients, the ps-Tg level, T stage, and response to initial RAIT are critical final outcome indicators.

## 1 Introduction

Although the incidence of differentiated thyroid cancer (DTC) in children and adolescents (caDTC) is low, it has been steadily increasing in recent years (1–3). Compared to adult thyroid cancer, caDTC has distinct differences in terms of its pathophysiological characteristics, clinical features, and long-term prognosis (4–7). Consequently, guidelines and treatment strategies designed for adult thyroid cancer patients are not fully applicable to children and adolescents. The American Thyroid Association (ATA) published its inaugural guidelines for the diagnosis and treatment of pediatric thyroid nodules and DTC in 2015, aiming to standardize the management of caDTC patients (8).

The primary approach to treating caDTC consists of total thyroidectomy, succeeded by adjuvant radioiodine therapy (RAIT) as deemed necessary (9, 10). The substantial benefits of RAIT, including a reduction in the recurrence rate and improvement in overall survival, have been documented among caDTC patients classified as having the highest risk (5, 11). The objective remains to preserve the current low disease-specific mortality rates observed in caDTC patients while minimizing therapy-related complications and the risk of overtreatment (8, 12).

Preablation-stimulated thyroglobulin (ps-Tg) levels have demonstrated a favorable predictive value for therapeutic outcomes and overall survival in the management of adult DTC patients (4, 5, 13, 14). Conversely, the prognostic utility of ps-Tg in caDTC patients has yet to be conclusively determined. Within this clinical context, the objective of our study was to examine the role of certain risk factors, such as ps-Tg, the ATA risk classification, and the therapeutic response to initial RAIT, in predicting the final disease status.

## 1 Materials and methods

### 2.1 Patients

The thyroid cancer databases from Shanghai Tenth People’s Hospital and the Affiliated Hospital of Qingdao University were retrospectively screened to identify all caDTC patients (aged ≤18 years) who received at least one standardized RAIT between January 2004 and December 2022. This study adhered to the principles of the Declaration of Helsinki and was approved by the Ethics Review Committee of the Affiliated Hospital of Qingdao University.

The inclusion criteria were as follows: 1) history of total thyroidectomy; 2) pathological type confirmed as DTC; 3) age ≤18 years at the time of initial RAIT; 4) receipt of standardized RAIT; 5) initial recurrence risk stratified as intermediate or high risk (**Supplementary Table 1**) (8); and 6) negativity for anti-thyroglobulin antibody (TgAb).The exclusion criteria were 1) incomplete regular follow-up data and 2) other malignant tumors or other antitumor treatments (such as chemotherapy, external radiation therapy, or targeted drug therapy.).

All selected caDTC patients were restaged according to the 8th edition of the American Joint Committee on Cancer (AJCC)/TNM staging system (15). Moreover, the assignment of the initial recurrence risk category adhered to the 2015 ATA pediatric risk stratification system (8).

The main clinical characteristics, which included gender, preoperative thyroid stimulating hormone (TSH) level, age at the initial RAIT, histological type, tumor size, multicentricity, extrathyroidal invasion, T stage, lymph node involvement (**Supplementary Table 2**), and recurrence risk, were documented. Additionally, the ps-Tg, TgAb, and details regarding RAIT administration (total amount of RAIT received and total number of RAIT sessions) were also recorded.

### 2.2 RT protocol and follow-up

Patients followed a low-iodine diet and ceased levothyroxine intake for 2-3 weeks to increase their TSH levels above 30 mIU/L (16, 17). Prior to RAIT administration, routine diagnostic procedures, including neck ultrasound and diagnostic whole body scan (Dx-WBS), were conducted (8). For prepubertal patients, the dose administered in each RAIT session was an empirical dosage of 1.0-1.5 mCi/kg (16). After puberty, a single dose of 100-200 mCi was administered based on the patient’s disease condition (9).

The therapeutic response to initial RAIT was assessed between 6 and 12 months after the initial RAIT according to the 2015 ATA therapeutic response classification, which categorizes responses into four distinct classes: excellent response (ER), biochemical incomplete response (BIR), structural incomplete response (SIR), and indeterminate response (IDR) (**Supplementary Table 3**) (4, 8). Subsequently, BIR and SIR were categorized into the incomplete response (IR) group, while ER and IDR were classified into the excellent or indeterminate response (EIDR) group. This reclassification was based on a combination of biochemical (TSH, ps-Tg, suppressed thyroglobulin (sup-Tg), and TgAb) and imaging findings (neck ultrasound, CT, MRI, Dx-WBS and any additional imaging exams) (7).

All patients were intervals of 6-12 months thereafter based on the individual patient’s risk and the clinical progression of the disease. Repeated RAIT was administered at least 1 year after the latest treatment session, contingent upon adequate ^131^I absorption by the lesions and a favorable clinical response (16, 18).

### 2.3 Definitions of the clinical outcomes

At the last visit, patients were categorized as either having EIDR or exhibiting IR, as determined by integrating laboratory findings and imaging results. IR was defined as sup-Tg ≥1 ng/mL or stimulated thyroglobulin (sTg) ≥10 ng/mL, or an upward trend in TgAb, with or without the presence of structural or functional lesions (16, 19, 20). Conversely, patients who did not meet the above criteria for IR at the last follow-up were classified as having EIDR.

### 2.4 Statistical analysis

Statistical analyses were performed using IBM SPSS software version 26.0. Categorical variables are represented herein by frequencies and percentages, while continuous variables are described as the mean ± SD or *M (P_25,_ P_75_)*. A receiver-operating characteristic (ROC) curve was established to predict the efficacy of the RAIT using ps-Tg, identifying the optimal diagnostic threshold. The Mann-Whitney *U* test, independent sample t test or chi-square test were performed for univariate analyses as necessary, and significant factors were then included in the logistic regression analysis to identify the independent risk factors that affect the efficacy of the RAIT. A p value less than 0.05 was considered to indicate statistical significance.

## 3 Results

### 3.1 Description of caDTC patient’s characteristics

The clinical characteristics of the 143 eligible caDTC patients are summarized in **Table 1**. The ratio of males to females was 1: 2.18, and the median age was 16 years at the time of the initial RAIT. PTC accounted for 96.5% of all patients, and the size of the primary tumor was 2.5 (1.5, 3.6) cm. Multicentricity of tumor lesions was present in 86 cases (60.1%), and extrathyroidal invasion was observed in 66 cases (46.2%). Thirty-three patients (23.1%) were reclassified into the T4 stage according to the 8th edition. Most patients (85.3%) were classified as N1b. By the 2015 ATA pediatric risk stratification, 75 patients (52.4%) were categorized into the intermediate-risk group, and 68 (47.6%) were classified into the high-risk group. The preoperative TSH level was 2.5 ± 1.0 mIU/L, and the ps-Tg level was 19.0 (7.6, 72.0) ng/mL. All patients underwent RAIT1-5 times, with a median total ^131^I activity of 150 mCi. The median duration of follow-up was 42.9 months (range 6.3-113.4 months).

**Table 1:** Clinical characteristics of the caDTC patients.

Characteristics	Patients n(%)	Median (IQR)	Range
Age (years)		16 (14, 17)	6-18
Gender			
Male	45 (31.5)		
Female	98 (68.5)		
Histological type			
PTC	138 (96.5)		
FTC	5 (3.5)		
Multicentricity			
Yes	86 (60.1)		
No	57 (39.9)		
Tumor size (cm)		2.5 (1.5, 3.6)	0.5-5.0
Extrathyroidal invasion			
Yes	66 (46.2)		
No	77 (53.8)		
T stage			
T1-3	110 (76.9)		
T4	33 (23.1)		
N stage			
N0-1a	21 (14.7)		
N1b	122 (85.3)		
Recurrence risk			
Intermediate	75 (52.4)		
High	68 (47.6)		
PreoperativeTSH (mIU/L)		2.5 ± 1.0	0.4-6.1
ps-Tg (ng/mL)		19.0 (7.6, 72.0)	2.1-17468.0
Total number of RAIT sessions		1 (1, 2)	1-5
Total ^131^I activity (mCi)		150 (100, 300)	50-760
Follow-up duration (months)		42.9 (20.7, 62.9)	6.3-113.4

caDTC, children and adolescents patients with differentiated thyroid cancer; PTC, papillary thyroid cancer; FTC, follicular thyroid cancer; RAIT, radioiodine therapy; T, tumor; N, node; TSH, thyroid stimulating hormone; ps-Tg, preablation stimulated thyroglobulin.

### 3.2 Therapeutic efficacy of RAIT

**Figure 1:**
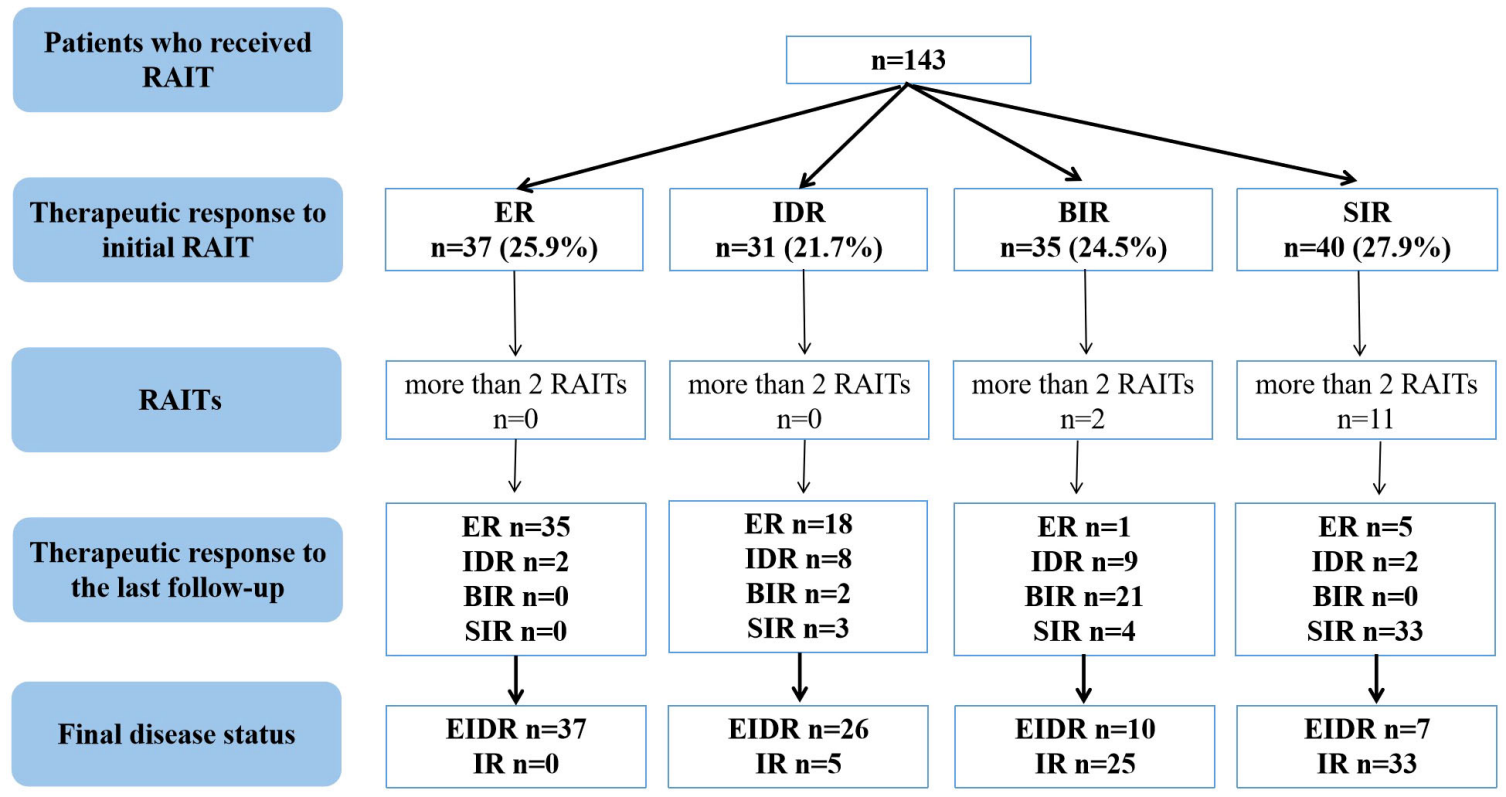
Flowchart of the therapeutic efficacy evaluation of RAIT in caDTC patients. caDTC, differentiated thyroid cancer in children and adolescents; RAIT, radioiodine therapy; ER, excellent response; IDR, indeterminate response; BIR, biochemical incomplete response; SIR, structural incomplete response; EIDR, excellent or indeterminate response; IR, incomplete response.

As shown in **Figure 1**, 37 (25.9%) caDTC patients had an ER, 31 (21.7%) had an IDR, 35 (24.5%) had a BIR, and 40 (27.9%) had an SIR at the therapeutic response to initial RAIT. Thus, after the initial RAIT, 47.6% (68/143) of patients were classified as having an EIDR, while 52.4% (75/143) were classified as having an IR. After a median follow-up of 42.9 months (range 6.3-113.4 months), EIDR was reported in 80 patients (55.9%), while IR was observed in the remaining 63 patients (44.1%). No deaths were observed among the patients at the last follow-up. Overall, of the 63 patients with an IR at the last follow-up, 23 had a BIR, and the remaining 40 had an SIR.

In this study, 13 caDTC patients with distant metastases (DM) underwent 3-5 RAITs for a total of 420-760 mCi of ^131^I, and showed SIR at the last follow-up (**Figure 1**). Among 35 caDTC patients with a BIR after the initial RAIT, only four had structural recurrence (2 patients had lymph node metastasis, and two patients had lung metastasis). Two patients with lung metastases underwent three and five RAIT sessions for a total of 450 and 760 mCi of ^131^I, respectively, and showed disease progression at the last follow-up. However, among patients with an SIR after the initial RAIT, three RAIT sessions were conducted for 7 patients, four RAIT sessions were conducted for 3 patients, and five RAIT sessions were conducted for 1 patient. Ultimately, 3 patients achieved partial response, 4 patients presented stable disease, and the remaining 4 patients exhibited progressive disease.

### 3.3 Predictive value of ps-Tg in patients with an IR at the last follow-up

**Figure 2:**
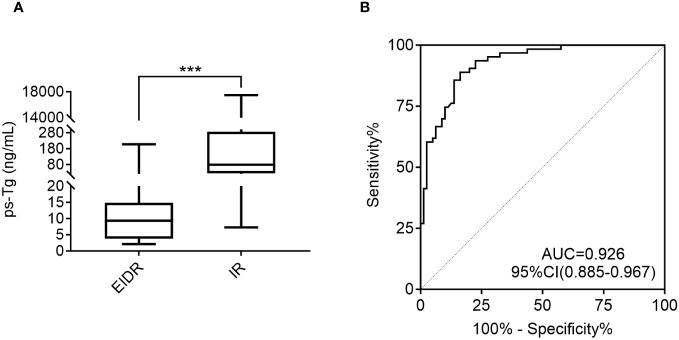
**(A)** Comparison of the ps-Tg levels in the EIDR and IR groups. **(B)** ROC curves of the ps-Tg test for detecting IR at the last follow-up. ps-Tg, preablation stimulated thyroglobulin; EIDR, excellent or indeterminate response; IR, incomplete response; ROC, receiver operating characteristic; AUC, area under the ROC curve; *** P <0.001.

The ps-Tg level in the EIDR group was 9.3 (3.8, 14.9) ng/mL, which was significantly lower than the 79.2 (25.1, 286.0) ng/mL in the IR group (**Figure 2A**). Furthermore, ROC analysis revealed that ps-Tg ≥20 ng/mL was the best threshold for discriminating IR from EIDR at the last follow-up, with a sensitivity of 88.9%, specificity of 83.8%, and AUC of 0.926 (95% CI: 0.885-0.967) (**Figure 2B**).

### 3.4 Univariate and multivariate analyses for the prediction of IR at the last follow-up

Univariate analysis revealed that patients with a larger tumor size (P=0.001), FTC (P=0.015), multicentricity (P=0.035), extrathyroidal invasion (P=0.003), stage T4 (P=0.000), stage N1b (P=0.003), a high recurrence risk (P=0.000), ps-Tg ≥20 ng/mL (P=0.000), IR as the therapeutic response to initial RAIT (P=0.000), a greater total number of RAIT sessions (P=0.000), and greater total ^131^I activity (P=0.000) had a higher probability of IR (**Table 2**). However, univariate analysis revealed no significant associations between IR and gender, age, or preoperative TSH level. According to multivariate analysis, only ps-Tg (as continuous variable and dichotomized with 20 ng/mL) (odds ratio (OR): 8.333, 95% confidence interval (CI): 2.143-32.400, P = 0.002), therapeutic response to initial RAIT categories (OR: 7.552, 95% CI: 1.780-32.038, P = 0.006), and T stage (OR: 4.202, 95% CI: 1.132-15.596, P = 0.032) were significantly associated with the risk of IR at the last follow-up (**Table 3**).

**Table 2:** Univariate analyses for characteristics of IR in caDTC.

Characteristics	n	EIDR group	IR group	χ^2^/*t*/U	P value
Gender				2.293^a^	0.130
Male	45	21(46.7%)	24(53.3%)		
Female	98	59(60.2%)	39(39.8%)		
Histological type				4.437^a^	0.015
PTC	138	80(58.0%)	58(42.0%)		
FTC	5	0(0%)	5(100%)		
Multicentricity				4.421^a^	0.035
Yes	86	42(48.8%)	44(51.2%)		
No	57	38(66.7%)	19(33.3%)		
Extrathyroidal invasion				9.090^a^	0.003
Yes	66	28(42.4%)	38(57.6%)		
No	77	52(67.5%)	25(32.5%)		
T stage				20.997^a^	0.000
T1-3	110	73(66.4%)	37(33.6%)		
T4	33	7(21.2%)	26(78.8%)		
N stage				8.851^a^	0.003
N0-N1a	21	18(85.7%)	3(14.3%)		
N1b	122	62(50.8%)	60(49.2%)		
Recurrence risk				19.351^a^	0.000
Intermediate	75	55(73.3%)	20(26.7%)		
High	68	25(36.8%)	43(63.2%)		
Preoperative TSH (mIU/L)		2.4 ± 0.9	2.7 ± 1.0	-1.322^b^	0.188
Age		16(14, 17)	16(13, 17)	2254.00^c^	0.273
Tumor size (cm)		2.0(1.3, 3.2)	3.0(2.2, 4.2)	1716.00^c^	0.001
Total RAIT times		1(1, 1)	2(1, 2)	1224.00^c^	0.000
Total ^131^I activity (mCi)		100(80, 150)	270(150, 350)	1039.00^c^	0.000
Ps-Tg (ng/mL)				74.477^a^	0.000
<20.0	74	67(90.5%)	7(9.5%)		
≥20.0	69	13(18.8%)	56(81.2%)		
Therapeutic response to initial RAIT				70.864^a^	0.000
IR	75	17(22.7%)	58(77.3%)		
EIDR	68	63(92.6%)	5(7.4%)		

caDTC, differentiated thyroid cancer in children and adolescents; EIDR, excellent or indeterminate response; IR, incomplete response; PTC, papillary thyroid cancer; FTC, follicular thyroid cancer; T, tumor; N, node; ps-Tg, preablation-stimulated thyroglobulin; TSH, thyroid stimulating hormone; RAIT, radioiodine therapy; ^a^means chi-squared test; ^b^means independent sample t test; ^c^means Mann-Whitney U test.

**Table 3:** Multivariate logistic regression analyses for the prediction of IR in caDTC patients.

Characteristics	P value	OR	95% CI
Histological type (PTC/FTC)	0.894	0.781	0.125-5.160
Tumor size (cm)	0.715	1.094	0.675-1.775
Multicentricity	0.218	0.432	0.114-1.642
Extrathyroidal invasion	0.199	2.673	0.596-11.998
T stage (T1-3/T4)	**0.032**	4.202	1.132-15.596
N stage (N0-N1a/N1b)	0.158	3.725	0.601-23.100
Recurrence risk (intermediate/high)	0.133	0.307	0.066-1.433
Ps-Tg (ng/mL) (<20/≥20)	**0.002**	8.333	2.143-32.400
Therapeutic response to initial RAIT (EIDR/IR)	**0.006**	7.552	1.780-32.038
Total RAIT times	0.942	1.082	0.128-9.144
Total ^131^I activity (mCi)	0.717	1.002	0.989-1.016

caDTC, differentiated thyroid cancer in children and adolescents; EIDR, excellent or indeterminate response; IR, incomplete response; PTC, papillary thyroid cancer; FTC, follicular thyroid cancer; T, tumor; N, node; ps-Tg, preablation stimulated thyroglobulin; RAIT, radioiodine therapy. Data in bold (p value <0.05) indicate statistical significance.

**Figure 3:**
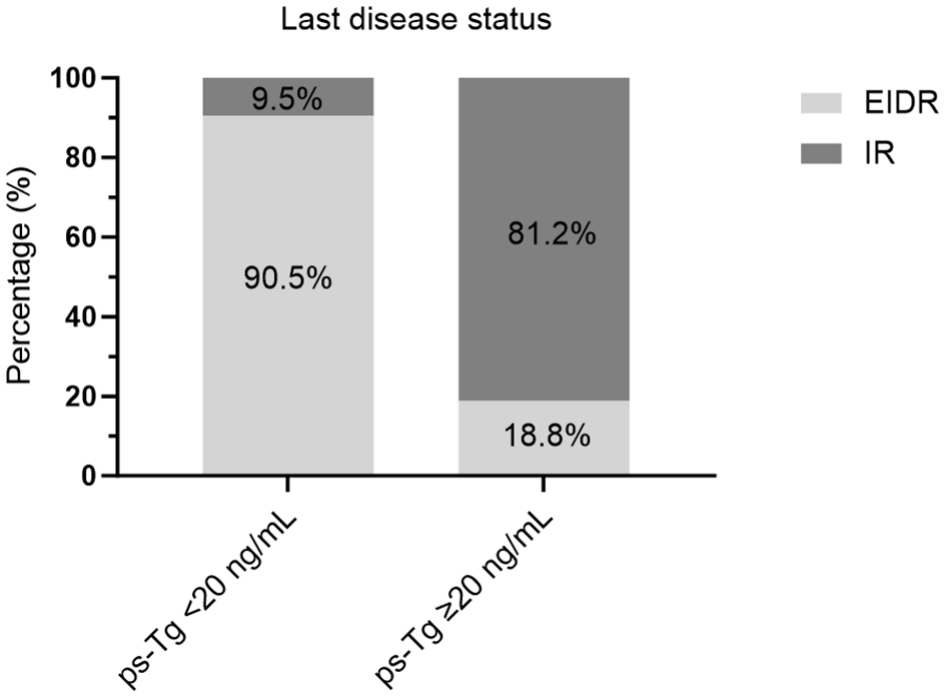
Evaluation of the last disease status in caDTC patients with high or low ps-Tg levels. ps-Tg, preablation stimulated thyroglobulin; EIDR, excellent or indeterminate response; IR, incomplete response.

**Figure 4:**
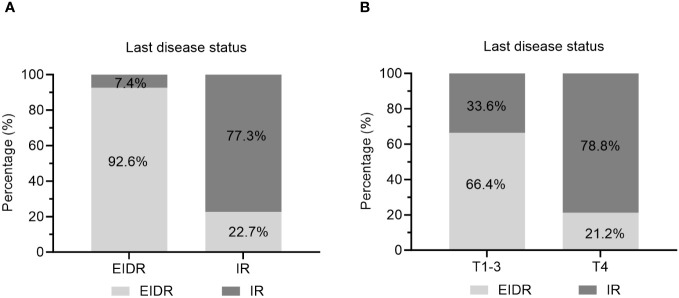
Evaluation of the last disease status in caDTC patients with **(A)** EIDR or IR after initial RAIT and **(B)** T1-3 or T4 disease. EIDR, excellent or indeterminate response; IR, incomplete response; T, tumor.

In particular, 81.2% of caDTC patients with baseline psTg≥20 ng/mL showed an IR, while only 9.5% of patients with psTg<20 ng/ml exhibited an IR at the end of follow-up (P<0.001) (**Figure 3**). Furthermore, among patients with EIDR after the initial RAIT, only 7.4% had an IR, while among patients with an IR after the initial RAIT, 77.3% had an IR at the end of follow-up (P<0.001) (**Figure 4A**). Moreover, 78.8% of patients with T4 disease showed an IR, while only 33.6% of patients with T1-3 disease showed an IR at the end of follow-up (P<0.001) (**Figure 4B**).

## 4 Discussion

RAIT, serving as a pivotal postoperative adjuvant treatment for caDTC patients, can significantly improve the overall survival rate of patients, and reduce their risk of recurrence, metastasis, and death (7–9, 21). In this retrospective study, we compiled data from two centers in China. Despite covering an extended period from 2004 to 2022 and including diverse geographical locations, we adhered to uniform international guidelines for the management of caDTC patients, allowing for the sharing and analysis of these data.

At the last follow-up, approximately 44.1% (63/143) of patients were assessed for real-time dynamics as having progressed to an IR. As a primary finding, we demonstrated that the ps-Tg could predict the ultimate efficacy of RAIT with an optimal cutoff threshold established at 20 ng/ml. In this context, ps-Tg appears to be the principal predictive marker of disease response, having a significantly greater impact on risk assessment than does the risk classification of the 2015 pediatric ATA guidelines (8). Moreover, we found that high ps-Tg levels, T4 stage, and IR to the initial RAIT were significantly associated with adverse outcomes.

Patients who exhibit an ER to initial RAIT generally have favorable prognoses, with lower rates of recurrence and higher survival probabilities (22). Conversely, those with an IR, particularly an SIR, face a greater risk of adverse outcomes (7, 8). Sung et al. (19) demonstrated that individuals in the indeterminate and incomplete response groups face a greater risk of recurrent or persistent disease than those in the an excellent response group. In the present study, approximately 47.6% (68/143) of caDTC patients were evaluated as having an EIDR to their initial RAIT. Moreover, the therapeutic response to the initial RAIT emerged as a critical determinant of long-term outcomes. Patients demonstrating an EIDR to initial RAIT exhibited markedly lower rates of IR (**Figure 4A**), emphasizing the importance of early and effective disease control. Our findings align with those reported by Cistaro et al. (5), which showed in a cohort of 276 caDTC patients who consecutively underwent total thyroidectomy and RAIT that the 1-year treatment response category was independently associated with the final disease status.

Tg serves as a highly specific and sensitive biomarker for detecting the presence of follicular thyroid cells (7, 12). The measurement of serum Tg is pivotal in the clinical management of DTC patients, and is widely regarded as the most sensitive technique for identifying the persistence or recurrence of the disease (4, 23–25). A meta-analysis conducted by Webb et al. (13), which included nearly 4,000 adult patients with DTC, demonstrated that with a threshold of 10 ug/mL, the negative predictive value of ps-Tg for predicting long-term disease remission reached an impressive 94%. This finding suggested that patients with low ps-Tg levels have a favorable prognosis and a reduced rate of tumor recurrence. Our investigation reaffirms that elevated ps-Tg levels are significantly associated with IR, which is consistent with previous reports in adult populations but tailored to the pediatric context (26, 27). A ps-Tg level of ≥20 ng/mL enhances the risk of recurrence, facilitating tailored therapeutic approaches and vigilant follow-up protocols.

Our investigation highlights T4 staging as a critical independent risk factor for RAIT efficacy in caDTC patients. Specifically, patients classified as having T4 disease exhibited substantially worse outcomes following RAIT than those classified as having T1-3 disease (**Figure 4B**). This finding aligns with the literature, which suggests that advanced T staging typically correlates with diminished treatment responses, particularly in RAIT, due to the aggressive nature and extensive local invasion characteristic of the T4 stage (7, 8, 28). Moreover, Wang et al. (29) quantitatively assessed the correlation between T4 stage and survival metrics such as overall survival (OS) and disease-specific survival (DSS). These studies confirm that T4 staging adversely affects survival outcomes, underscoring the urgency of adopting tailored therapeutic strategies for patients with higher T stages. Similarly, a study by Li et al. (30) revealed that the T stage significantly influences the prognosis of stage IV B DTC patients. In caDTC patients, the T4 stage is often associated with a greater tumor burden and increased local invasiveness, which may impede the absorption and effectiveness of iodine-131. Additionally, patients at this stage are likely to have higher recurrence rates and poorer long-term survival outcomes, necessitating more aggressive and personalized treatment approaches in clinical practice.

TSH is a growth factor that influences the initiation and progression of DTC. However, the relationship between preoperative TSH levels and DTC remains controversial. Previous studies have demonstrated that elevated preoperative TSH concentrations are associated with an increased risk of thyroid malignancy (31, 32). A multicenter retrospective study by Aihong Mao et al. (33), which included 1,997 patients with papillary thyroid microcarcinoma (PTMC), suggested that the preoperative TSH concentration should be considered a risk predictor for tumor progression in PTMC patients. In contrast, in this study, we found no significant difference in preoperative TSH levels between patients in the EIDR group and those in the IR group (2.4 ± 0.9 vs. 2.7 ± 1.0 mIU/L, P=0.188), confirming that the preoperative TSH level is not a predictive factor for RAIT efficacy in caDTC patients.

Therefore, we suggest that higher ps-Tg levels, T4 stage, and IR to initial RAIT might predict higher IR rates in caDTC patients receiving RAIT. For patients with these high-risk factors, a single RAIT session may be insufficient to achieve optimal efficacy. A comprehensive assessment of the patient’s condition is necessary, and a more aggressive or multimodal treatment strategy should be considered. This strategy may include more frequent monitoring, higher RAIT doses, combined treatments (such as surgery, external radiotherapy or targeted therapy), and close follow-up. By identifying these high-risk factors, clinicians can develop more flexible treatment plans, avoid a one-size-fits-all approach, and truly achieve personalized treatment. For low-risk patients (such as ps-Tg<20 ng/mL, T1-3 staging, and the response to initial RAIT as EIDR), taking TSH suppression therapy and reducing the intensity and frequency of RAIT can minimize treatment-related side effects and improve quality of life.

Our study is subject to several limitations, First, although we strictly followed the inclusion and exclusion criteria to select the samples, selection bias might still have existed because of the small number of patients eventually included. Second, given the typically prolonged survival of pediatric patients, a longer follow-up period is necessary to fully understand the long-term outcomes and to provide a more comprehensive analysis. Third, there was heterogeneity of the management and follow-up approaches, considering the long period of nearly 20 years in the two research centers. Therefore, further multicenter or larger cohort-based and extended observation period studies are required to corroborate our results.

## 5 Conclusion

In conclusion, our study provides compelling evidence that multiple factors influence RAIT efficacy in caDTC patients, with ps-Tg levels, T stage, and the therapeutic response to initial RAIT serving as key prognostic indicators. These insights underscore the importance of a tailored, risk-adapted approach for managing caDTC, paving the way for enhanced therapeutic strategies and improved patient outcomes.

## Data availability statement

The original contributions presented in the study are included in the article/**Supplementary Material**. Further inquiries can be directed to the corresponding authors.

## Ethics statement

The studies involving humans were approved by Ethical Committee of the Affiliated Hospital of Qingdao University. The studies were conducted in accordance with the local legislation and institutional requirements. Written informed consent for participation in this study was provided by the participants’ legal guardians/next of kin.

## Author contributions

CW: Conceptualization, Data curation, Formal analysis, Funding acquisition, Investigation, Methodology, Project administration, Software, Writing – original draft. YL: Data curation, Formal analysis, Investigation, Methodology, Writing – original draft. GW: Data curation, Investigation, Writing – original draft. XL: Writing – review & editing. YZ: Data curation, Investigation, Writing – original draft. CL: Data curation, Investigation, Writing – original draft. JL: Data curation, Investigation, Writing – original draft. NH: Data curation, Writing – original draft. ZW: Data curation, Investigation, Writing – original draft. ZS: Data curation, Investigation, Writing – original draft. FL: Writing – review & editing. GL: Data curation, Writing – original draft. RW: Conceptualization, Formal analysis, Investigation, Methodology, Writing – review & editing. XW: Conceptualization, Writing – review & editing.
