# Supplementary material for: Prognostic factors in children and adolescents with differentiated thyroid cancer treated with total thyroidectomy and radioiodine therapy: a retrospective two-center study from China
Source: Front Endocrinol (Lausanne). 2024 Jul 22;15:1419141. doi: 10.3389/fendo.2024.1419141 (PMC11298371; doi:10.3389/fendo.2024.1419141)
Supplement: Supplementary file 1 [file Table_1.docx]

**Prognostic factors in children and adolescents with differentiated thyroid cancer** **treated with total thyroidectomy and radioiodine therapy: A retrospective two-center study from China**

*Congcong Wang^1,^* †*, Yutian Li^2,^* †*, Guoqiang Wang^1^, Xinfeng Liu^1^, Yingying Zhang^1^, Chenghui Lu^1^, Jiao Li^1^, Na Han^1^, ZengHua Wang^1^, Zengmei Si^1^, Fengqi Li^1^, Gaixia Lu^3^, Renfei Wang^3,*^ , Xufu Wang^1,*^*

^1^ Department of Nuclear Medicine, The Affiliated Hospital of Qingdao University, No.16 Jiangsu Road, Shinan District, Qingdao, Shandong 266003, China.

^2^ Department of Radiology, Qingdao Women and Children's Hospital, No.217 Liaoyang West Road, Shibei District, Qingdao, Shandong 266000, China.

^3^ Department of Nuclear Medicine, Shanghai Tenth People’s Hospital, Tongji University School of Medicine, Shanghai 200072, China.

† Congcong Wang and Yutian Li contributed equally to this work.

**Corresponding Authors:**

^*^ Xufu Wang; Department of Nuclear Medicine, The Affiliated Hospital of Qingdao University, No.16 Jiangsu Road, Shinan District, Qingdao, Shandong 266003, China. E-mail: [wangxufu@sina.com](mailto:wangxufu@sina.com);

^*^ Renfei Wang; Department of Nuclear Medicine, Shanghai Tenth People’s Hospital, Tongji University School of Medicine, Shanghai 200072, China. E-mail: [roslyn_en@163.com](mailto:roslyn_en@163.com)

**Running title:** Prognostic factors of caDTC RAIT

**Supplemental Table 1:** Definition of the initial risk stratification of caDTC patients.

| Initial risk stratification | Definition (1, 2) |
| --- | --- |
| Low risk | Disease grossly confined to the thyroid (T1–T3) with N0/Nx disease or with microscopic N1a disease (≤5 and <0.2 cm) |
| Intermediate risk | Extensive N1a disease (＞5 or ≥0.2 cm) or minimal N1b disease (≤10 at the lateral neck and <3 cm) |
| High risk | Extensive N1b disease (＞10 at the lateral neck or ≥3 cm) or locally invasive disease (T4) or distant metastasis |

Abbreviations: caDTC=children and adolescents patients with differentiated thyroid cancer; T=tumor; N=node.

**Supplemental Table 2**: Definition of TNM, multicentricity, lymph node involvement, and extrathyroidal invasion in patients with caDTC.

|  | Definition (3) |
| --- | --- |
| Primary tumor (T) | The extent of the primary tumor |
| Tx | Primary tumor cannot be assessed |
| T0 | No evidence of primary tumor |
| T1 | ≤2 cm, limited to the thyroid |
| T2 | > 2 cm but ≤ 4 cm, limited to the thyroid |
| T3 | > 4 cm, limited to the thyroid, or gross extrathyroidal extension invading only the strap muscles (sternohyoid, sternothyroid, thyrohyoid, or omohyoid muscles) |
| T4 | Includes gross extrathyroidal extension into major neck structures (gross extrathyroidal extension invading the subcutaneous soft tissues, larynx, trachea, esophagus, recurrent laryngeal nerve, or prevertebral fascia or encasing the carotid artery or mediastinal vessels from a tumor of any size) |
| Lymph node (N) involvement | Tumor cells pass through the wall of the lymphatic canal, follow the lymphatic flow to the location of the lymph node, and produce invasive growth of the tumor in the lymph node. |
| Nx | Regional lymph nodes cannot be assessed |
| N0 | No metastatic nodes |
| N1a | Metastasis to level VI or VII (pretracheal, paratracheal, or prelaryngeal/Delphian or upper mediastinal) lymph nodes. This can be unilateral or bilateral disease. |
| N1b | Metastasis to unilateral, bilateral, or contralateral lateral neck lymph nodes (Levels I, II, III, IV, or V) or retropharyngeal lymph nodes |
| Distant metastasis (DM) |  |
| M0 | No distant metastasis |
| M1 | Distant metastasis |
| Multicentricity | The presence of two or more primary cancer foci in the thyroid gland |
| Extrathyroidal invasion | Tumor extending beyond the thyroid capsule to invade the subcutaneous soft tissues, larynx, trachea, esophagus, or recurrent laryngeal nerve, or prevertebral fascia, or encasing the carotid artery or mediastinal vessels. |

Abbreviations: caDTC=children and adolescents patients with differentiated thyroid cancer; T=tumor; N=node.

**Supplemental Table 3**: Definition of the modified dynamic risk stratification by response to initial RAIT in caDTC patients.

| Response to therapy category | Definition (1, 4) |
| --- | --- |
| Excellent response (ER) | Stimulated thyroglobulin (sTg) < 1 ng/mL or suppressed thyroglobulin (sup-Tg) <0.2 ng/mL and TgAb negative and negative imaging |
| Indeterminate response (IDR) | 0.2 ng/mL≤Sup-Tg<1 ng/mL or 1 ng/mL≤sTg<10 ng/mL or detectable TgAb levels (stable/declining) and no evidence of structural/functional disease |
| Biochemical incomplete response (BIR) | Sup-Tg ≥1 ng/mL or sTg ≥10 ng/mL or rising TgAb levels and negative imaging |
| Structural incomplete response (SIR) | Structural or functional evidence of disease regardless of the thyroglobulin or TgAb levels |

Abbreviations: caDTC=children and adolescents patients with differentiated thyroid cancer.

**References**

1. Francis GL, Waguespack SG, Bauer AJ, Angelos P, Benvenga S, Cerutti JM, et al. Management Guidelines for Children with Thyroid Nodules and Differentiated Thyroid Cancer. Thyroid (2015) 25(7):716-759. doi:10.1089/thy.2014.0460
2. Sung TY, Jeon MJ, Lee YH, Lee YM, Kwon H, Yoon JH, et al. Initial and Dynamic Risk Stratification of Pediatric Patients With Differentiated Thyroid Cancer. J Clin Endocrinol Metab (2017) 102(3):793-800. doi:10.1210/jc.2016-2666
3. Perrier ND, Brierley JD, Tuttle RM. Differentiated and anaplastic thyroid carcinoma: Major changes in the American Joint Committee on Cancer eighth edition cancer staging manual. CA Cancer J Clin (2018) 68(1):55-63. doi:10.3322/caac.21439
4. Haugen BR, Alexander EK, Bible KC, Doherty GM, Mandel SJ, Nikiforov YE, et al. 2015 American Thyroid Association Management Guidelines for Adult Patients with Thyroid Nodules and Differentiated Thyroid Cancer: The American Thyroid Association Guidelines Task Force on Thyroid Nodules and Differentiated Thyroid Cancer. Thyroid (2016) 26(1):1-133. doi:10.1089/thy.2015.0020
